# An Evolution of Shoulder Periprosthetic Infections Management: MicroDTTect, Bioactive Glass and Tantalum Cones Employment

**DOI:** 10.3390/jcm9113683

**Published:** 2020-11-16

**Authors:** Alfonso Maria Romano, Tiziana Ascione, Pasquale Casillo, Guglielmo Nastrucci, Massimiliano Susanna, Angelo Di Giunta, Francesco Ascione

**Affiliations:** 1 Department of Shoulder Surgery, Campolongo Hospital, 84127 Salerno, Italy; alfonso.maria.romano@gmail.com (A.M.R.); pasquale.casillo87@gmail.com (P.C.); guglielmonastrucci@hotmail.it (G.N.); 2 Department of Orthopaedic and Traumatology Surgery, Ospedale Buon Consiglio Fatebenefratelli, 80123 Napoli, Italy; 3 Department of Infectious Diseases, A. Cardarelli Hospital, 80131 Napoli, Italy; tizianascione@hotmail.com; 4 Orthopedic and Traumatology Unit, San Donà di Piave Hospital, 30027 Venezia, Italy; massimiliano.susanna@gmail.com; 5 Orthopaedic Division of Policlinico “G.B. Morgagni”, 95125 Catania, Italy; adigiunta@yahoo.com

**Keywords:** shoulder arthroplasty, prosthesis, reverse, infection, complication, bone loss, humeral, tantalum

## Abstract

Periprosthetic joint infections of the shoulder (PJIS) are the major cause for revision within the first two post-operative years, and are challenging both to diagnose and treat. Success depends on early identification of microorganisms, appropriate surgical procedures and efficient antibiotic administration. The peculiar microbiology of the shoulder may render the criteria for hip/knee PJI management inappropriate. In addition, later cases with clinically subtle signs often present diagnostic challenges. In recent years, specific issues of PJIS have been managed through the use of new instruments, such as MicroDTTect in pathogen detection and Bioactive Glass and tantalum cones in humeral bone loss. In the literature to date, no reports have been found that discuss the application in shoulder revisions and infections. The early identification of the microorganisms that cause infection may help improve both treatment strategies and the efficacy of therapy. MicroDTTect proved to be more efficient than swab collection for bacterial identification in orthopedic surgery, thus reducing analysis costs. The increasing number of shoulder arthroplasties is associated with an increase in the number of revisions. In cases of massive metaphyseal humeral bone loss, several techniques have been described; no reports have been reported regarding tantalum in humeral bone loss management. In some cases the tantalum cones required adaptation for femoral diaphysis in the augmentation of the humerus metaphysis and bone loss management improvement. Obtaining stable osseointegration of prosthetic implants is one of the greatest issues in orthopedic surgery, and even more crucial in revisions. Bioactive glasses demonstrated good regenerative and osseointegration properties, and an excellent candidate as a bone graft, scaffold and antibiotics deliverer. The Bioactive glasses were used to increase prosthesis-bone interface stability and fill bone defects in PJIS revision surgeries, contributing to the prevention of re-infection. Longer-term follow-up will be necessary to determine if construction durability is improved in the long term.

## 1. Introduction

Periprosthetic joint infection of the shoulder (PJIS) is a rare but serious complication that is challenging to treat. Success depends on early identification of microorganisms, appropriate surgical procedures, and efficient antibiotic administration.

The mean incidence of PJIS is reported as 1.1% [1], and reverse arthroplasty (RSA) infection rate 3.8% (reaching 10% in the subgroup of young male patients) [2,3]. Pottinger et al. [4] reported a detection of PJIS in 56% of 193 shoulder prosthesis revisions. They therefore suggested that every report of pain, stiffness, and loosening of the shoulder prosthesis should be regarded as an indication of infection, until proven otherwise.

PJIS is the major cause for revision within the first two post-operative years after an arthroplasty [5,6,7,8]. Treatment options for PJIS include intravenous antibiotics, tissue debridement with retention of the prosthesis, resection arthroplasty, one-stage and two-stage exchange procedures, arthrodesis, and amputation.

There has not been evidence from the literature to establish clear standardized concepts for diagnosis or surgical and antibiotic treatment [9]. PJIS status should always be excluded or proven preoperatively and not during surgery, but since the establishment of biofilm around the implant may render the empirical broad spectrum antibiotic treatment useless, intraoperative tests are necessary to confirm the preoperative diagnosis by obtaining at least two concordant cultures [10].

The peculiar microbiology of the shoulder may render the diagnostic criteria for hip/knee PJIS and its management inappropriate. Furthermore, later cases with clinically subtle signs often present diagnostic challenges [11,12].

All patients with suspected PJIS should receive perioperative antibiotics at the time of the revision surgical procedure. Cefazolin is the agent most likely to provide optimal tissue concentration for prophylaxis against the three most common causative organisms [13].

Treatment strategy depends on infection timing: within 30 days after surgery, a surgical debridement with polyethylene exchange (and glenosphere in RSA) may be appropriate [14]. In cases of hematogenous infection 30 days or more after surgery, implant removal with tissues debridement, one-stage or two-stage procedure (followed by species-directed antibiotic administration), should be considered [10,14,15]. Finally, in chronic infections in less serious cases or in patients who are ineligible for revision, surgical debridement with implant removal, antibiotic spacer placement, or simple resection arthroplasty would be the treatment of choice.

PJIS are commonly treated through a two-stage procedure as this is the solution that provides a compromise between reliable eradication of the infection and satisfactory post-surgery functional outcome, especially in those with low virulence infection [16].

It is essential for the surgeon to evaluate these crucial aspects and plan for a surgical revision that ensures biomechanical implant stability and functionality after infection, whilst taking into consideration the risk of recurrent infection and the high rate of postoperative complication.

The aim of the present study was to review new opportunities in shoulder periprosthetic infections, focusing on the use of MicroDTTect in pathogen detection, Bioactive Glass, and tantalum cones in humeral bone loss, and to report on our experience of utilizing them.

## 2. Bacteria Identification—The MicroDTTect

The early identification of microorganisms that cause infection may help improve treatment strategies and efficacy, increasing implant healing rate, reducing the severity of sequelae and improving quality of life.

Biofilm-related infections and low-grade infections need to be considered in cases of doubtful infection, as they tend to be accompanied by few signs of inflammation. The isolation and identification of bacteria when embedded in biofilm is difficult as they are poorly recognized by the immune system and as a result, infection becomes chronic.

Consequently, several techniques have been developed for the detection of microorganisms in order to overcome the drawback of biofilm-related infections. Sonication of the removed implants has been shown to be more sensitive than conventional tissue cultures, especially in patients who were treated with antibiotics prior to surgery [17]. The limitations of this method that have become evident are the need for dedicated laboratory tools, the intrinsic risk of contamination, the size of the explanted prostheses, and bacteria proliferation in the sonication bath water. To remedy these limitations, treatment of the prosthesis with dithiothreitol (DTT) solution has been proposed.

DTT is a strong reducing agent commonly used in clinical, chemistry and microbiology laboratories, which also acts as a protein denaturant. Moreover, it may enhance the detachment of bacteria from biofilms on arthroplasties, thus reducing staphylococcal biofilm. Previous studies have shown that DTT treatment can substitute sonication in the microbiological diagnosis of PJIS; they are easier to use, they do not require any specific laboratory instruments, and are more sensitive than sonication in *Staphylococcus epidermidis*, which is often involved in PJIS [18].

Thus, over the last three years we have pursued the standardized use of MicroDTTect technology in shoulder arthroplasty revisions, specifically in the collection and transportation of explanted prostheses for bacteria identification.

The MicroDTTect device is used during the operation, directly placing the explanted specimens inside the device that is employed for the transportation and processing of samples, from the operating theatre to the microbiology laboratory, improving bacterial culture under safe and sterile conditions. The system contains a specific concentration of DTT that can dislodge bacteria from the biofilm adhering to prosthetic surfaces.

Periprosthetic tissue culture has generally been considered the reference standard for the identification of the pathogens involved in PJIS, although such cultures lack sufficient sensitivity (described as ranging from 70% to 90%) and specificity (ranging from 67% to 91%) [19]. MicroDTTect proved superior to swab collection for bacterial identification in orthopaedic surgery, with 80% circa sensitivity when analyzing explanted prostheses, osseosynthetic devices, and biological tissues [20].

Sambri et al. [21], however, found no difference in sensitivity between DTT and sonication for detection of periprosthetic infections, and both tests were more sensitive than standard tissue cultures, in a comparative randomized study. The DTT system remains quick and simple to use and may offer reduced costs and processing time in comparison to sonication.

## 3. Infection Prevention and Osseointegration—Bioactive Glass

Obtaining stable osseointegration of prostheses implants is an even greater issue in infected revised arthroplasties; the deposition of bioactive coatings on the implant surface to be in contact with the bone may be a valuable strategy in favoring “physiological” osseointegration [22], whilst preventing reinfection. Bioactive glasses are a new generation of bioceramics designed for bone grafting and skeletal regenerative therapies, and have been proven to fill a bone defect, subsequently being gradually replaced by functional tissue.

These biomaterials exhibit significantly higher bone regenerative properties, due to increased surface area and porosity [23]. One of the most important properties of bioactive glasses is their efficacy against the most common Gram-positive and Gram-negative bacteria, creating a bacteria-free environment whilst allowing healing and regeneration of the defect area. They also possess ordered mesoporous structure, making them excellent candidates for matrixes in drug delivery applications such as for antibiotics.

Polo et al. [24] demonstrated that bioactive glasses inhibit the proliferation of the bacteria cells, permitting levofloxacin release without cellular damage when levofloxacin was not present inside the pores, supporting the hypothesis that the cytotoxicity caused is due to the released levofloxacin and therefore against bacteria.

Bioactive glasses have a wide range of applications such as bone grafts, scaffolds, coating materials, and are used for treatment in cases of hypersensitivity. In vitro studies showed that the integrity of the implant is retained after immersion in biological fluids, which is of crucial importance in the safety of any clinical application [25].

The development of surfaces with low bacterial adhesion together with biocompatibility and antibiotic release properties may provide both a solution in the prevention of infections and in the treatment of infected arthroplasties.

**Figure 1:**
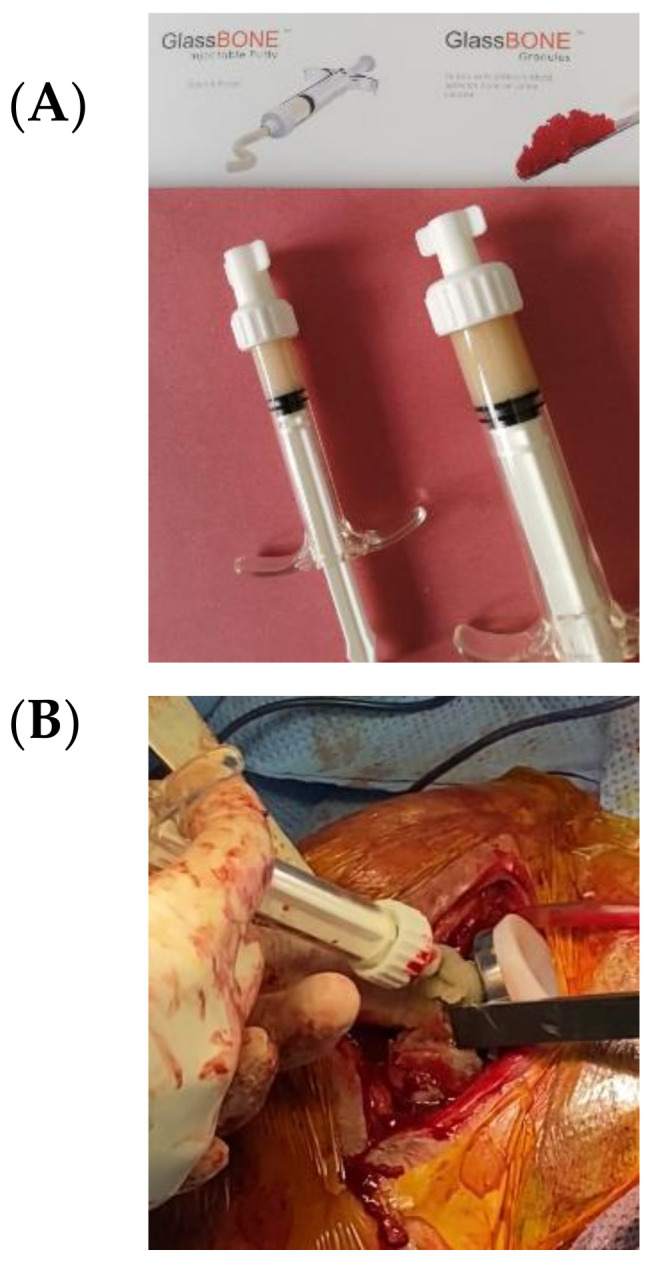
(**A**) The bone substitute GlassBONE, made of bioactive glass. (**B**) Ready-to-use, it can be injected through the syringe directly into the bone defect or onto the bone-prosthesis interface.

**Figure 2:**
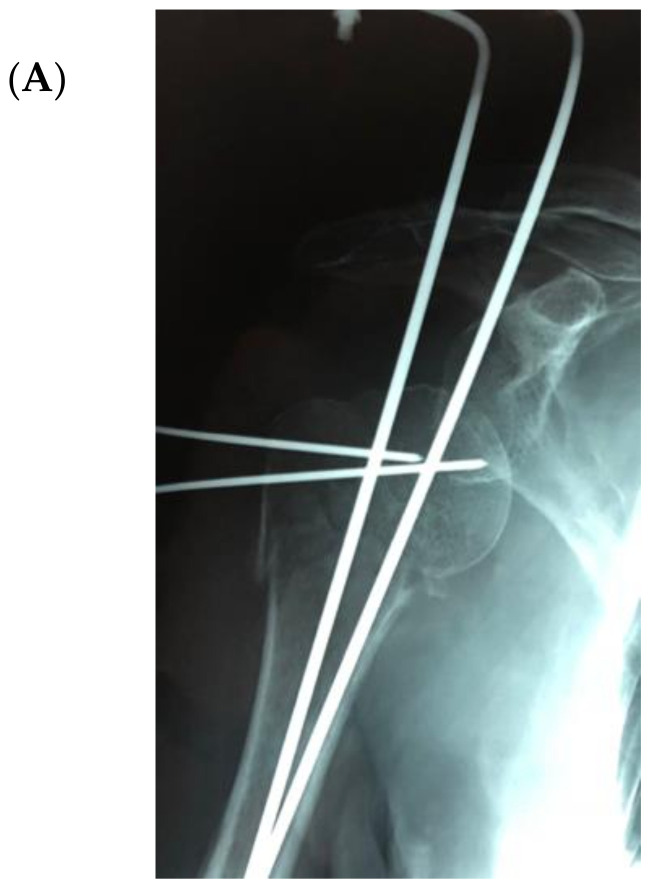

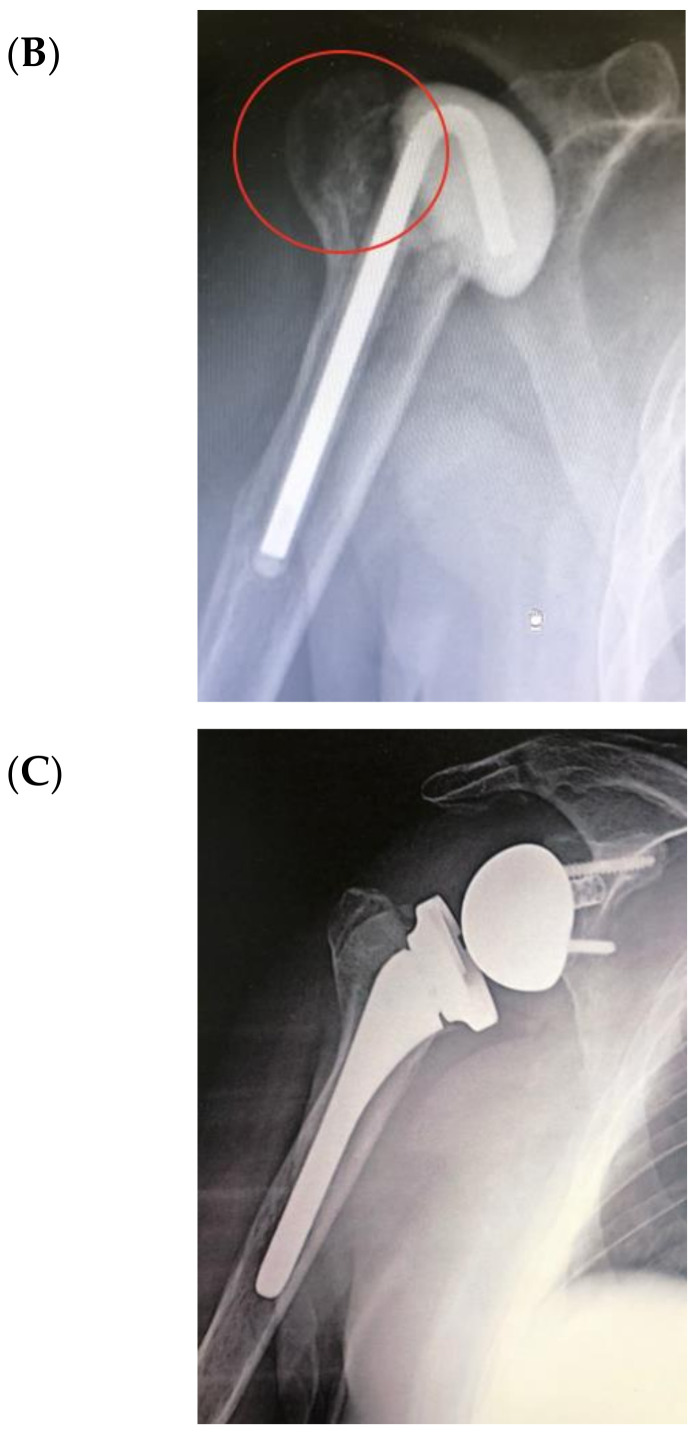
An infection after percutaneous pinning of a proximal humerus fracture. (**A**) Preoperative X-ray. (**B**) The subsequent implanting of a cement antibiotic spacer (red circle underlines bone loss); (**C**) the final reverse arthroplasty (RSA) with the addition of bioactive glass in the tuberosity defect.

The bone substitute GlassBONE Putty (Noraker, Villeurbanne, France), made of bioactive glass, was routinely employed in revision surgeries requiring bone loss filling in our surgeries. This ceramic is composed of Silicium, Calcium, Sodium and Phosphorous, minerals which are naturally present in the human body; it is in a ready-to-use format and can be injected through the syringe (Figure 1): it may be used both to increase prosthesis-bone interface stability and fill bone defects in PJIS revision surgeries, also contributing to preventing re-infection. In this regard, a peculiar case was reported (Figure 2): infection after percutaneous treatment of a proximal humerus fracture. The pinning removal and the implant of a cement antibiotic spacer were attempted. Finally, an RSA (Equinoxe Shoulder System, Exactech Inc., Bloomington, MN, USA) was implanted with the addiction of bioactive glass to prevent tuberosity defect augmentation and re-infection.

Although a conspicuous presence of in vitro studies were developed on the role of bioactive glasses promoting osseointegration and inhibiting bacteria proliferation, as well as some papers regarding cervical/lumbar spinal fusion surgeries, fracture nonunion treatment and arthrodesis employing bioactive glasses [26,27], to date, no clinical evidence reported their use in joint arthroplasty or revision and further studies are needed.

Another possible solution is silver-coating the prosthesis. Various in vitro and clinical studies have shown that silver coatings effectively inhibit or even prevent the formation of biofilms of various bacteria on knee and hip arthroplasty metal surfaces [28,29].

In a retrospective analysis of 34 patients, Zajonz et al. [30] demonstrated that the rate of reinfection of modular mega-endoprostheses on hip and knee joints can be reduced by the use of silver-coated implants. Reinfection time can also be delayed by utilizing silver-coated implants.

Argyria may be a complication of silver-coated implants. It is difficult to pinpoint the level at which silver may cause serious local or systemic damage, but most of the cases had no significant side effects from silver; trace elements of silver in the blood were often raised but below the toxic threshold [31].

However, no studies on silver-coated shoulder prosthesis have been carried out in revision surgery and it may be an interesting topic of further investigation, although the benefits would have to outweigh the economic outlay.

## 4. Bone Loss Management—Tantalum Cones

**Figure 3:**
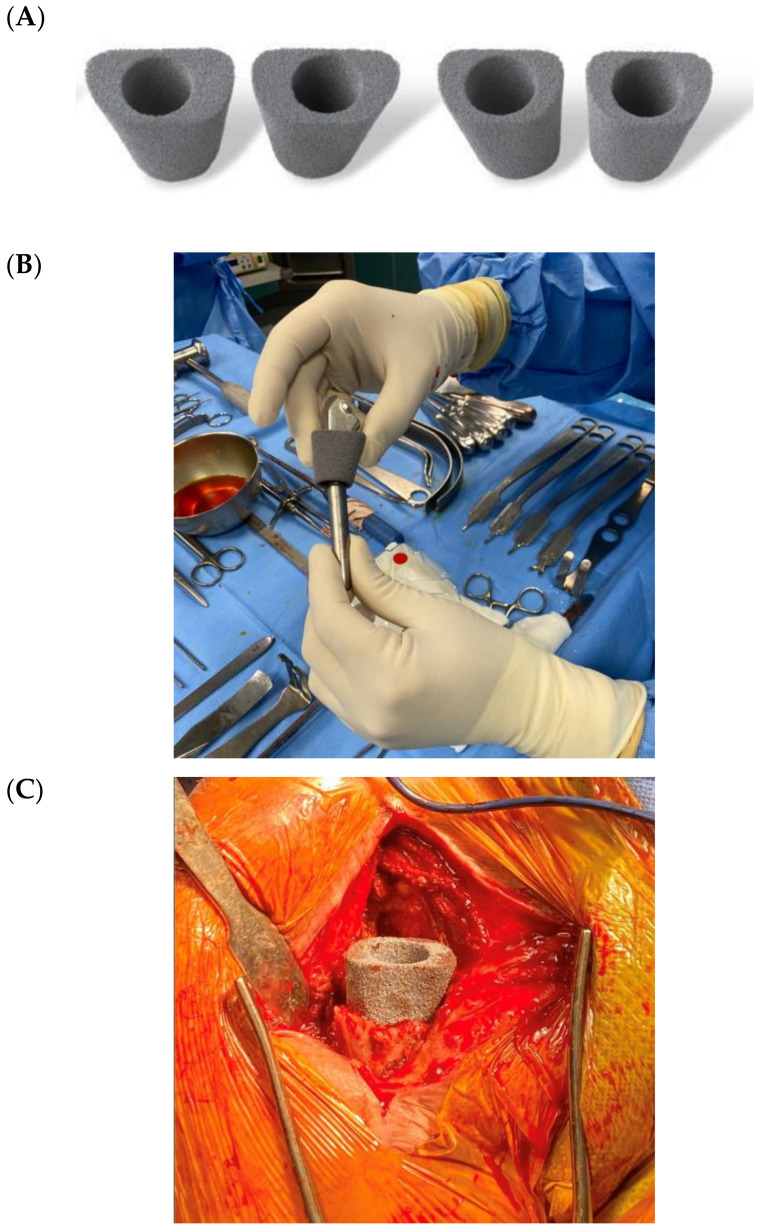
(**A**) Tantalum cones for femoral diaphysis defects in knee arthroplasty revision; (**B**) adaptation for a humeral stem and (**C**) augmentation in the humerus metaphysis.

The increase in the number of shoulder arthroplasties is associated with an increase in the number of revisions [32,33] and PJIS are the major cause for revision in the first two post-operative years [1]. Frequently, shoulder revision surgeries are due to humeral proximal bone defects, both as a consequence of infection and as a result of primary arthroplasty removal. In cases of massive metaphyseal humeral bone loss, several techniques have been described such as bone allografts or bone cement [34,35,36]. However, to date, there no studies in the literature that have reported the use of tantalum in humeral bone loss, whereas in hip/knee arthroplasty [37,38,39,40,41,42] or glenoid [43,44,45] revisions this has been extensively studied. Tantalum cones in femoral diaphysis were first employed in our revision surgeries in order to augment the humerus metaphysis and improve bone loss management (Figure 3).

The use of pre-molded porous tantalum implants is a technical option that may increase immediate resistance and, due to the porosity of this material, osseointegration and penetration of the cement is improved, thus reducing the risk of repeat loosening [38,39].

Tantalum shows excellent biocompatibility and unique characteristics that render it particularly well suited for bone ingrowth [33]. High friction coefficient, high porosity, and a stiffness similar to that of trabecular bone, reinforce the bone interface, thus obtaining biological fixation [42]. Recent studies have reported promising results with use of porous tantalum cones to address bone defects in revision knee arthroplasties [40,41]. However, a viable alternative is the “hybrid technique,” in which a cementless, diaphyseal-engaging stem with cement throughout the metaphysis and undersurface of the component is utilized. This technique demonstrated excellent 5- and 10-year survivorship in aseptic prosthetic revision [46,47]. A potential disadvantage may be represented by revision of well-fixed tantalum cones: explant can be very challenging with extended bone loss and the issue should be considered when using tantalum augments, but other treatment alternatives in humeral massive defects (tumoral prostheses, bone allografts or bone cement) are burdened by technical problems and major complications, as well [34,35,36].

**Figure 4:**
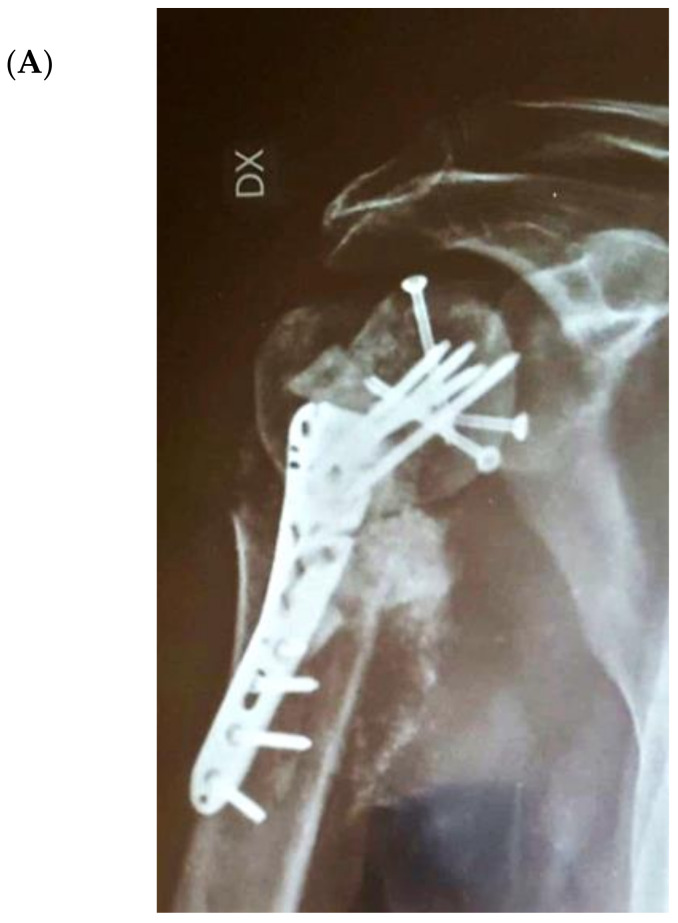

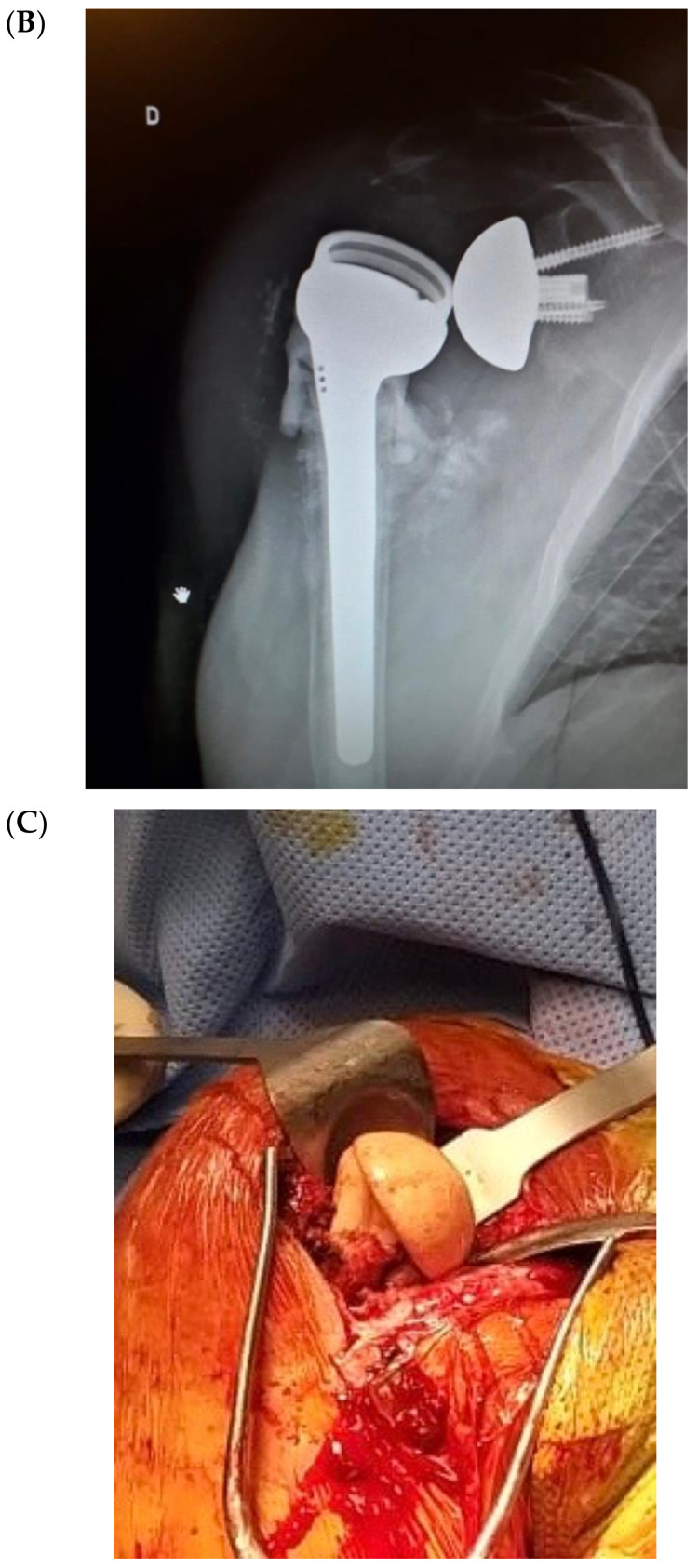

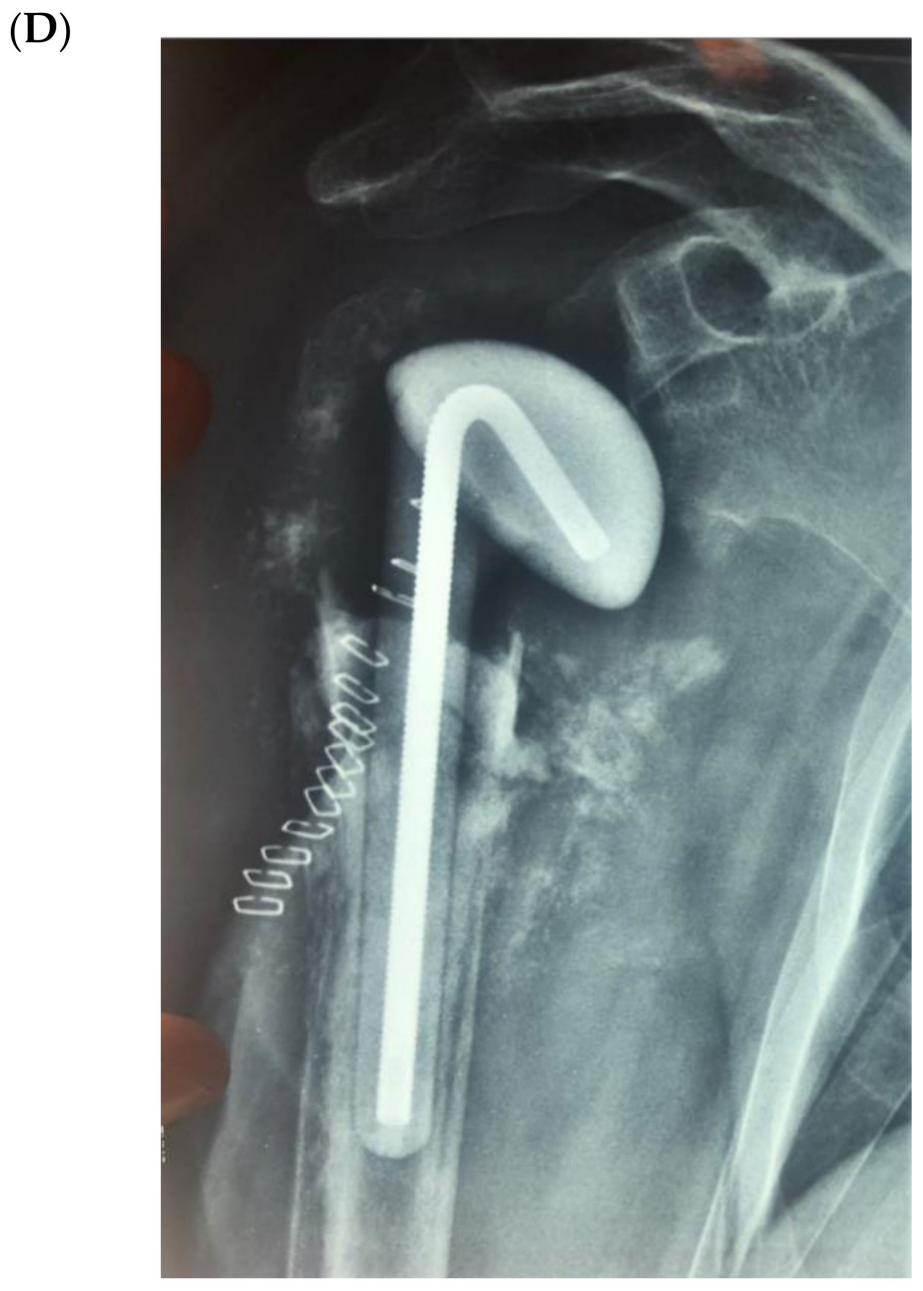
(**A**) The X-ray of a case of an infected proximal humerus fracture sequelae, originally treated with a plate. (**B**) After plate removal, the first RSA was implanted, which subsequently dislocated, resulting in infection. (**C**) A two-stage procedure was performed: removal of the RSA, debridement and implant of a cement antibiotic spacer with vancomycin. (**D**) Spacer X-ray postoperative image.

Figure 4 illustrates an infected case of proximal humerus fracture sequelae, originally treated with a plate, then treated according to our protocol. After plate removal, an RSA was implanted, which subsequently dislocated, resulting in infection. A two-stage procedure was performed: removal of the RSA, debridement, and implantation of a cement antibiotic spacer with vancomycin. MicroDTTect identified the pathogen (*Cutibacterium acnes*), intravenous antibiotics and infection eradication followed.

Similar to the cited hybrid technique used in knee revision, three procedures for shoulder arthroplasty revision with consistent metaphyseal bone loss were conducted, employing the bioactive glass and tantalum cones. Two of these were the result of two-stage surgeries after PJIS.

The senior shoulder surgeon (AMR) implanted an RSA using the Equinoxe Shoulder System (Exactech Inc., Bloomington, MN, USA). The humeral canal was sized and then compacted until rotational stability of the trials was achieved and a longer cemented stem was required. The glenosphere diameter was 42 mm.

**Figure 5:**
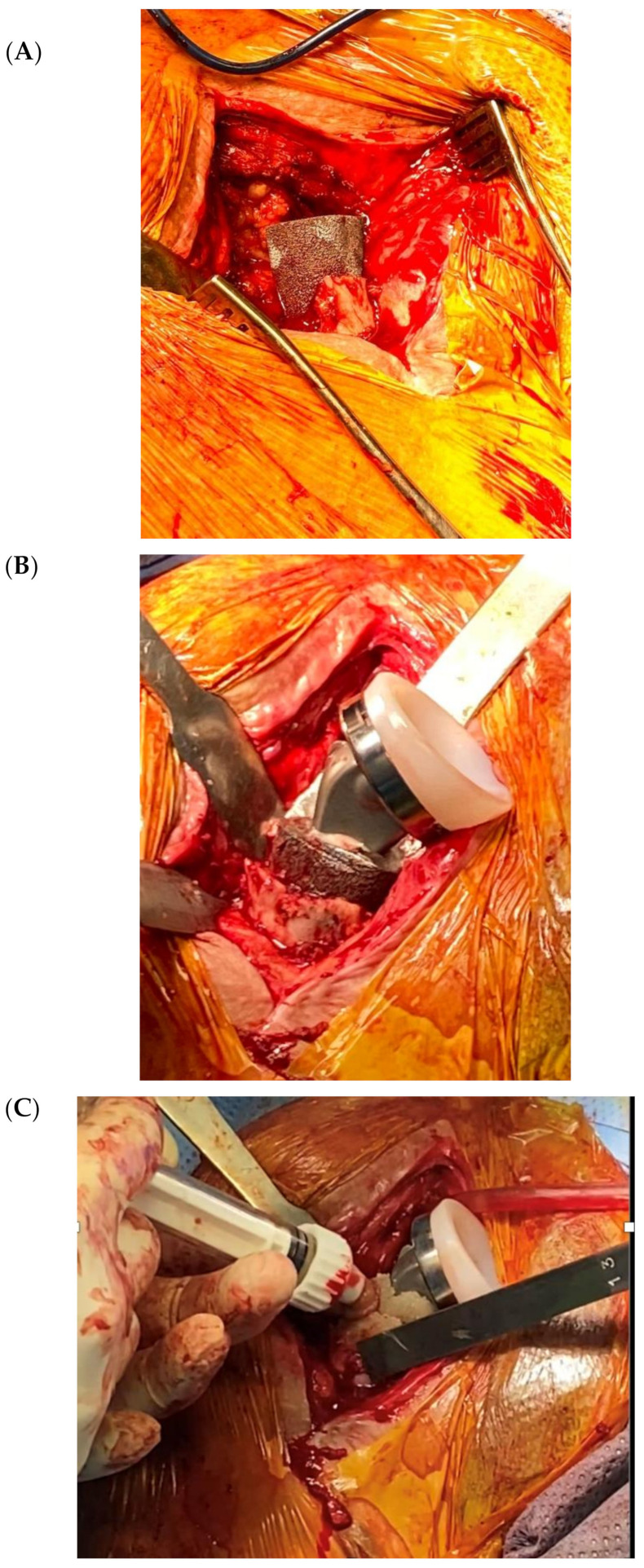

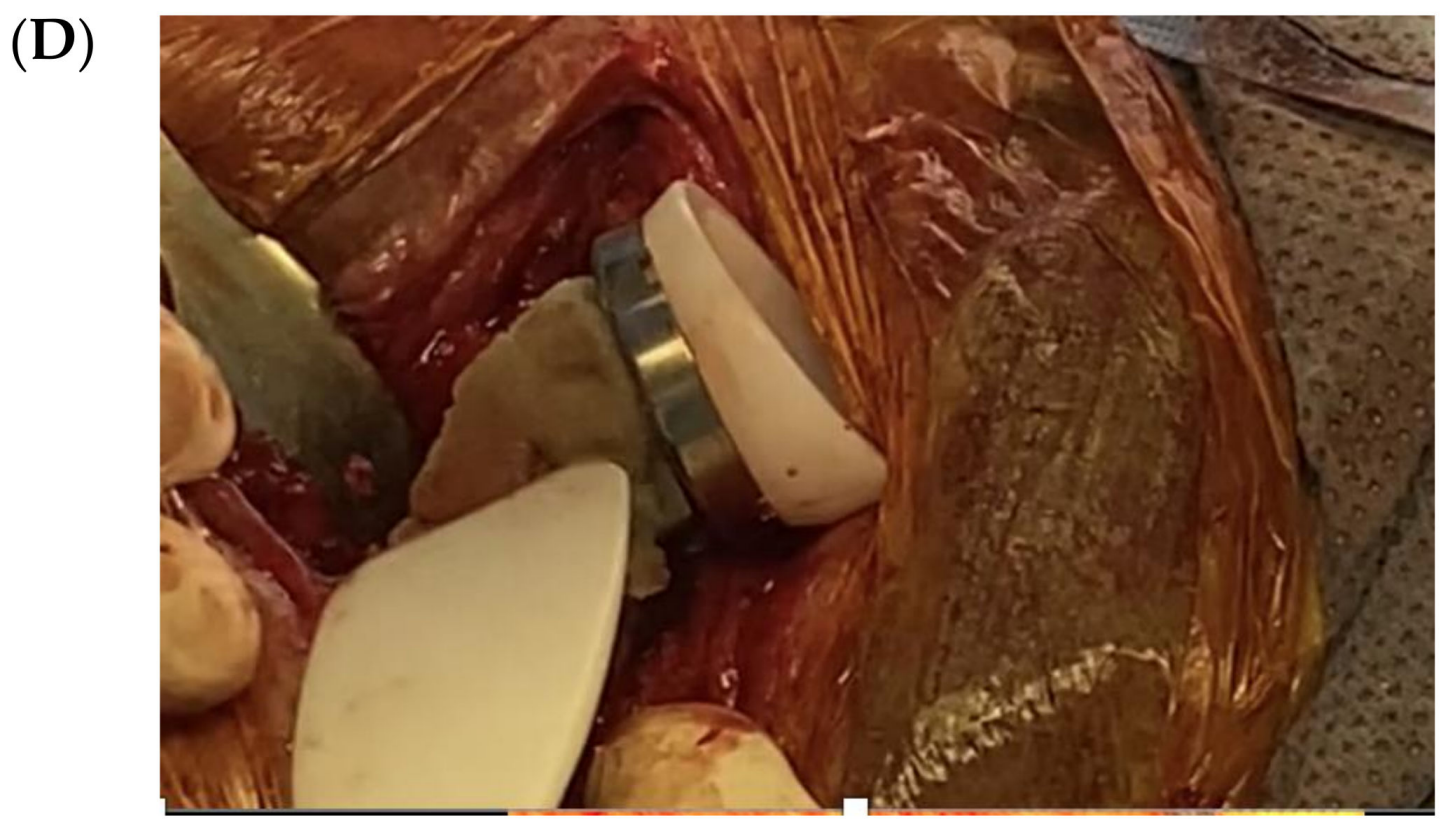
(**A**,**B**) In the second stage surgery, a femoral diaphysis tantalum cone was inserted in the proximal humerus with a cemented stem RSA. (**C**,**D**) Finally, bioactive glass proximal augmentation was performed.

**Figure 6:**
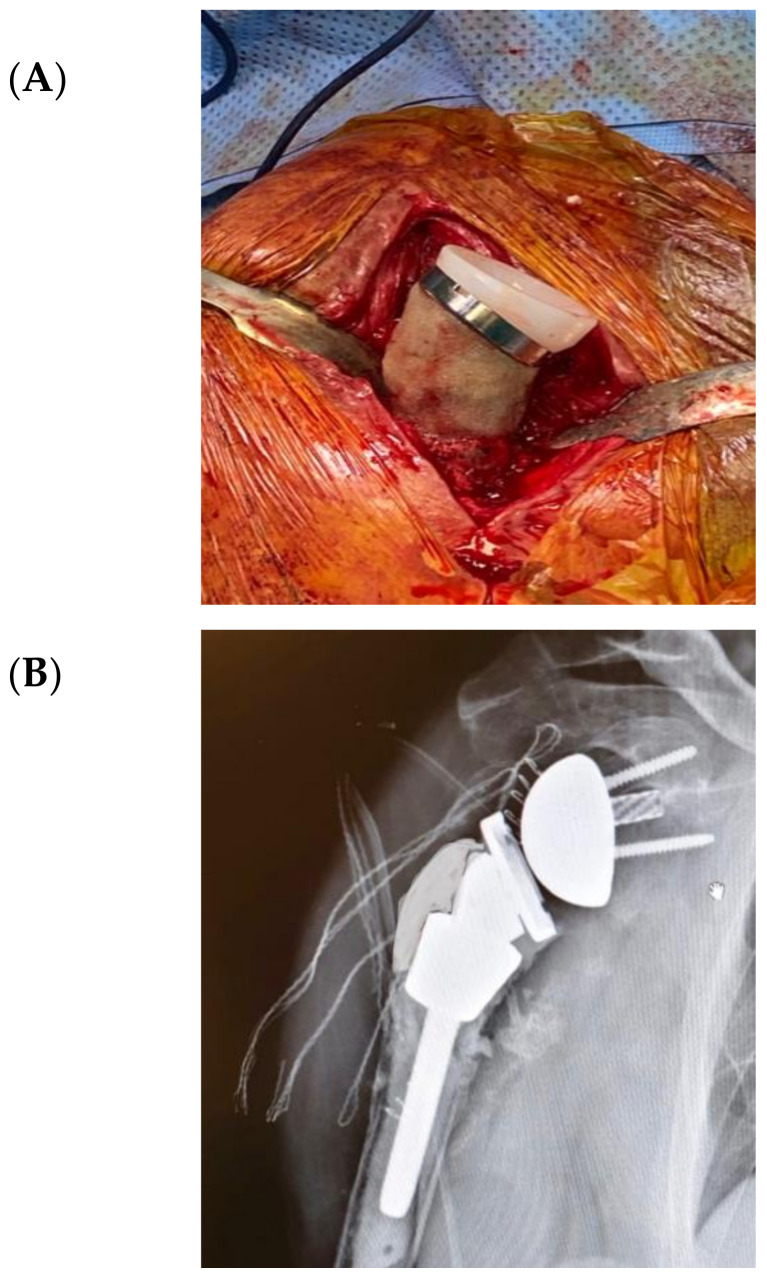
Revision final result. (**A**) Introperative image; (**B**) Postoperative X-Ray.

A porous tantalum cone (Zimmer, Warsaw, IN, USA) was employed for extensive defects of humeral metaphyseal bone. Bioactive glass was added both between the stem and cones, and as a proximal humerus augmentation (Figure 5 and Figure 6) in order to improve defect filling and attempt to avoid a new infection, thus increasing implant stability and integration. The surgical technique has already been described in detail [48]. The high porosity of these cones results in satisfactory primary fixation on recipient bone and cement fixation on the intramedullary side, sealing the component. Local filling of metaphyseo-diaphyseal bone defects with cones can limit the use of massive allografts or tumoral prostheses.

Further follow-up will be necessary to determine if construction durability improves in the long-term.

## 5. Conclusions

Although no clear guidelines have been defined in PJIS, the results of the instruments discussed regarding pathogen detection, prosthetic revision, and bone loss management are encouraging. Nevertheless, longer-term follow-up will be necessary in order to evaluate clinical outcomes, radiographic integration, and any complications that may arise.

## References

[1] Zumstein M.A., Pinedo M., Old J., Boileau P. (2011). Problems, complications, reoperations, and revisions in reverse total shoulder arthroplasty: A systematic review. J. Shoulder Elb. Surg..

[2] Farshad M., Gerber C. (2010). Reverse total shoulder arthroplasty—from the most to the least common complication. Int. Orthop..

[3] Ascione F., Bugelli G., Domos P., Neyton L., Godeneche A., Bercik M.J., Walch G. (2017). Reverse shoulder arthroplasty with a new convertible short stem: Preliminary 2- to 4-year follow-up results. J. Shoulder Elb. Arthroplast..

[4] Pottinger P., Butler-Wu S., Neradilek M.B., Merritt A., Bertelsen A., Jette J.L., Warme W.J., Matsen F.A. (2012). Prognostic factors for bacterial cultures positive for propionibacterium acnes and other organisms in a large series of revision shoulder arthroplasties performed for stiffness, pain, or loosening. JBJS.

[5] Portillo M.E., Salvadó M., Alier A., Sorli L., Martínez S., Horcajada J.P., Puig L. (2013). Prosthesis failure within 2 years of implantation is highly predictive of infection. Clin. Orthop. Relat. Res..

[6] Guarrella V., Chelli M., Domos P., Ascione F., Boileau P., Walch G. (2019). Risk factors for instability after reverse shoulder arthroplasty. Shoulder Elb..

[7] Neyton L., Erickson J., Ascione F., Bugelli G., Lunini E., Walch G. (2019). Grammont Award 2018: Scapular fractures in reverse shoulder arthroplasty (Grammont style): Prevalence, functional, and radiographic results with minimum 5-year follow-up. J. Shoulder Elb. Surg..

[8] Merolla G., Walch G., Ascione F., Paladini P., Fabbri E., Padolino A., Porcellini G. (2018). Grammont humeral design versus onlay curved-stem reverse shoulder arthroplasty: Comparison of clinical and radiographic outcomes with minimum 2-year follow-up. J. Shoulder Elb. Surg..

[9] Garrigues G.E., Zmistowski B., Cooper A.M., Green A., Abboud J., Beazley J., Bozhkova S., Brandao P., Chen A., Choon D. (2019). Proceedings from the 2018 International Consensus Meeting on Orthopedic Infections: Evaluation of periprosthetic shoulder infection. J. Shoulder Elb. Surg..

[10] Fink B., Sevelda F. (2017). Periprosthetic joint infection of shoulder arthroplasties: Diagnostic and treatment options. BioMed Res. Int..

[11] Ascione F., Braile A., Romano A.M., di Giunta A., Masciangelo M., Senorsky E.H., Samuelsson K., Marzano N. (2020). Experience-optimised fast track improves outcomes and decreases complications in total knee arthroplasty. Knee.

[12] Vasso M., Braile A., Ascione F., Toro G., De Cicco A., Lepore F., Schiavone Panni A. (2019). Two-stage reimplantation in periprosthetic knee infection. Eur. Rev. Med. Pharmacol. Sci..

[13] Schwarz E.M., Parvizi J., Gehrke T., Aiyer A., Battenberg A., Brown S.A., Callaghan J.J., Citak M., Egol K., Garrigues G.E. (2019). 2018 International Consensus meeting on musculoskeletal infection: Research priorities from the general assembly questions. J. Orthop. Res..

[14] Cooper M.E., Trivedi N.N., Sivasundaram L., Karns M.R., Voos J.E., Gillespie R.J. (2019). Diagnosis and management of periprosthetic joint infection after shoulder arthroplasty. JBJS Rev..

[15] Paxton E.S., Green A., Krueger V.S. (2019). Periprosthetic Infections of the Shoulder: Diagnosis and Management. J. Am. Acad. Orthop. Surg..

[16] Assenmacher A.T., Alentorn-Geli E., Dennison T., Baghdadi Y.M.K., Cofield R.H., Sánchez-Sotelo J., Sperling J.W. (2017). Two-stage reimplantation for the treatment of deep infection after shoulder arthroplasty. J. Shoulder Elb. Surg..

[17] Portillo M.E., Salvadó M., Alier A., Martínez S., Sorli L., Horcajada J.P., Puig L. (2014). Advantages of sonication fluid culture for the diagnosis of prosthetic joint infection. J. Infect..

[18] Drago L., Signori V., De Vecchi E., Vassena C., Palazzi E., Cappelletti L., Romanò D., Romanò C.L. (2013). Use of dithiothreitol to improve the diagnosis of prosthetic joint infections. J. Orthop. Res..

[19] Bergin P.F., Doppelt J.D., Hamilton W.G., Mirick G.E., Jones A.E., Sritulanondha S., Helm J.M., Tuan R.S. (2010). Detection of periprosthetic infections with use of ribosomal RNA-based polymerase chain reaction. J. Bone Jt. Surg. Am..

[20] Calori G.M., Colombo M., Navone P., Nobile M., Auxilia F., Toscano M., Drago L. (2016). Comparative evaluation of MicroDTTect device and flocked swabs in the diagnosis of prosthetic and orthopaedic infections. Injury.

[21] Sambri A., Cadossi M., Giannini S., Pignatti G., Marcacci M., Neri M.P., Maso A., Storni E., Gamberini S., Naldi S. (2018). Is treatment with dithiothreitol more effective than sonication for the diagnosis of prosthetic joint infection?. Clin. Orthop..

[22] Baino F., Minguella-Canela J., Korkusuz F., Korkusuz P., Kankılıç B., Montealegre M., De los Santos-López M., Vitale-Brovarone C. (2019). In vitro assessment of bioactive glass coatings on alumina/zirconia composite implants for potential use in prosthetic applications. Int. J. Mol. Sci..

[23] Alizadeh-Osgouei M., Li Y., Wen C. (2019). A comprehensive review of biodegradable synthetic polymer-ceramic composites and their manufacture for biomedical applications. Bioact. Mater..

[24] Polo L., Gómez-Cerezo N., Aznar E., Vivancos J.-L., Sancenón F., Arcos D., Vallet-Regí M., Martínez-Máñez R. (2017). Molecular gates in mesoporous bioactive glasses for the treatment of bone tumors and infection. Acta Biomater..

[25] El-Tablawy S.Y., Abd-Allah W.M., Araby E. (2018). Efficacy of irradiated bioactive glass 45S5 on attenuation of microbial growth and eradication of biofilm from AISI 316 L discs: In-vitro study. Silicon.

[26] Shi E., Carter R., Weinraub G.M. (2019). Outcomes of hindfoot arthrodesis supplemented with bioactive glass and bone marrow aspirate: A retrospective radiographic study. J. Foot Ankle Surg..

[27] Barrey C., Broussolle T. (2019). Clinical and radiographic evaluation of bioactive glass in posterior cervical and lumbar spinal fusion. Eur. J. Orthop. Surg. Traumatol..

[28] Alt V. (2017). Antimicrobial coated implants in trauma and orthopaedics–A clinical review and risk-benefit analysis. Injury.

[29] Wilding C.P., Cooper G.A., Freeman A.K., Parry M.C., Jeys L. (2016). Can a silver-coated arthrodesis implant provide a viable alternative to above knee amputation in the unsalvageable, infected total knee arthroplasty?. J. Arthroplast..

[30] Zajonz D., Birke U., Ghanem M., Prietzel T., Josten C., Roth A., Fakler J.K.M. (2017). Silver-coated modular Megaendoprostheses in salvage revision arthroplasty after periimplant infection with extensive bone loss—A pilot study of 34 patients. BMC Musculoskelet. Disord..

[31] Wyatt M.C., Foxall-Smith M., Roberton A., Beswick A., Kieser D.C., Whitehouse M.R. (2019). The use of silver coating in hip megaprostheses: A systematic review. HIP Int..

[32] Werner B.S., Ascione F., Bugelli G., Walch G. (2017). Does arm lengthening affect the functional outcome in onlay reverse shoulder arthroplasty?. J. Shoulder Elb. Surg..

[33] Giardella A., Ascione F., Mocchi M., Berlusconi M., Romano A.M., Oliva F., Maradei L. (2017). Reverse total shoulder versus angular stable plate treatment for proximal humeral fractures in over 65 years old patients. Muscles Ligaments Tendons J..

[34] Boileau P. (2016). Complications and revision of reverse total shoulder arthroplasty. Orthop. Traumatol. Surg. Res..

[35] Ascione F., Domos P., Guarrella V., Chelli M., Boileau P., Walch G. (2018). Long-term humeral complications after Grammont-style reverse shoulder arthroplasty. J. Shoulder Elb. Surg..

[36] Chalmers P.N., Boileau P., Romeo A.A., Tashjian R.Z. (2019). Revision reverse shoulder arthroplasty. J. Am. Acad. Orthop. Surg..

[37] Bohl D.D., Brown N.M., McDowell M.A., Levine B.R., Sporer S.M., Paprosky W.G., Della Valle C.J. (2018). Do porous tantalum metaphyseal cones improve outcomes in revision total knee arthroplasty?. J. Arthroplast..

[38] Boureau F., Putman S., Arnould A., Dereudre G., Migaud H., Pasquier G. (2015). Tantalum cones and bone defects in revision total knee arthroplasty. Orthop. Traumatol. Surg. Res..

[39] Levine B., Sporer S., Valle C., Jacobs J., Paprosky W. (2010). Porous tantalum in reconstructive surgery of the knee—A review. J. Knee Surg..

[40] Long W.J., Scuderi G.R. (2009). Porous tantalum cones for large metaphyseal tibial defects in revision total knee arthroplasty. J. Arthroplast..

[41] Meneghini R.M., Lewallen D.G., Hanssen A.D. (2008). Use of porous tantalum metaphyseal cones for severe tibial bone loss during revision total knee replacement. J. Bone Jt. Surg. Am..

[42] Welldon K.J., Atkins G.J., Howie D.W., Findlay D.M. (2008). Primary human osteoblasts grow into porous tantalum and maintain an osteoblastic phenotype. J. Biomed. Mater. Res. A.

[43] Sandow M., Schutz C. (2016). Total shoulder arthroplasty using trabecular metal augments to address glenoid retroversion: The preliminary result of 10 patients with minimum 2-year follow-up. J. Shoulder Elb. Surg..

[44] Ascione F., Routman H.D., Tashjian R.Z. (2019). Severe Glenoid Erosion (B2, B3, C, E2, E3) Treated with RSA. Complex and Revision Shoulder Arthroplasty.

[45] Ascione F., Kilian C.M., Laughlin M.S., Bugelli G., Domos P., Neyton L., Godeneche A., Edwards T.B., Walch G. (2018). Increased scapular spine fractures after reverse shoulder arthroplasty with a humeral onlay short stem: An analysis of 485 consecutive cases. J. Shoulder Elb. Surg..

[46] Edwards P.K., Fehring T.K., Hamilton W.G., Perricelli B., Beaver W.B., Odum S.M. (2014). Are cementless stems more durable than cemented stems in two-stage revisions of infected total knee arthroplasties?. Clin. Orthop. Relat. Res..

[47] Sah A.P., Shukla S., Della Valle C.J., Rosenberg A.G., Paprosky W.G. (2011). Modified hybrid stem fixation in revision TKA is durable at 2 to 10 years. Clin. Orthop. Relat. Res..

[48] Romano A.M., Oliva F., Nastrucci G., Casillo P., Giunta A.D., Susanna M., Ascione F. (2017). Reverse shoulder arthroplasty patient personalized rehabilitation protocol. Preliminary results according to prognostic groups. Muscles Ligaments Tendons J..

